# Risk Factors for Type 2 Diabetes Mellitus Relapse in More Than 5-Year Follow-up After Sleeve Gastrectomy with Transit Bipartition

**DOI:** 10.1007/s11695-025-07906-5

**Published:** 2025-05-13

**Authors:** Cüneyt Kırkıl, Mesut Yur, İlayda Aydın, Ahmet Bozdağ, Ahmet Aslan, Mehmet Fatih Ebiloğlu

**Affiliations:** 1 Private Medikal Hospital, Elazığ, Turkey; 2 Fırat University, Elazığ, Turkey; 3 Fethi Sekin City Hospital, Elazığ, Turkey

**Keywords:** Type 2 diabetes mellitus, Relapse, ABCD score, Transit bipartition

## Abstract

**Background:**

In some patients who achieved complete remission (CR) of type 2 diabetes mellitus (T2DM) after sleeve gastrectomy with transit bipartition (TB), T2DM relapses after a while. ABCD scoring predicts the likelihood of remission following TB. However, the factors affecting T2DM relapse are unknown.

**Methods:**

The data of patients with CR after TB who were followed for more than 5 years was analyzed retrospectively.

**Results:**

The median follow-up of 56 patients, 29 of whom were female (51.8%), was 71 months (range: 61 to 101). Eleven of 56 patients (19.6%) had relapse in T2DM. Patients with an ABCD score less than 4 had a significantly higher rate of relapse. Its sensitivity and specificity rates were 90.9% and 93.3%, respectively. Preoperative C-peptide level (OR 0.032 [CI 0.003–0.295], *p* = 0.002), LDL-cholesterol level (OR 1.025 [CI 1.005–1.045], *p* = 0.013), duration of T2DM (OR 1.553 [1.216–1.983], *p* < 0.001), ABCD score (OR 0.047 [0.006–0.361], *p* = 0.003), and FIB-4 index (OR 6.073 [1.496–24.656], *p* = 0.012) were risk factors.

**Conclusions:**

Patients with longer durations of T2DM, higher LDL-cholesterol levels, lower C-peptide levels and ABCD scores, and worse liver fibrosis are at a higher risk of relapse after achieving initial CR of T2DM with TB.

## Introduction

Type 2 diabetes mellitus (T2DM) remission rate is higher after metabolic bariatric surgery (MBS) than with non-surgical treatment. Previous studies have suggested that the optimal clinical response rate of MBS varies between 15.5 and 94.6% for various procedures [1]. Various scoring systems, like ABCD (age, BMI, C-peptide, diabetes duration), IMS (Individualized Metabolic Score), and DiaRem (diabetes remission) scores, have been developed to predict the likelihood of T2DM remission after MBS [2]. Although it is known that patients with metabolic dysfunction-associated steatotic liver disease (MASLD) have a twice as high risk of acquiring diabetes [3], the impact of MASLD on T2DM remission was not taken account of by any of the scoring methods.

Since the clinical response rates of various surgical procedures in achieving T2DM remission vary widely, it is reasonable to assume that scoring systems will vary in their ability to predict T2DM remission according to the surgical models used. Despite being a relatively new and uncommon MBS procedure, sleeve gastrectomy with transit bipartition (TB) is increasingly being used in certain countries [4]. Recently, we have shown that the ABCD score is successful in predicting the probability of T2DM remission after TB [5]. However, the results of TB throughout a follow-up period longer than 5 years have not yet been reported in the literature.

Even though it is disappointing, the reality that some patients experience relapse after achieving T2DM remission is unavoidable. Unfortunately, one-third of patients with initial remission later suffer from relapse [6, 7]. It is a matter of curiosity not only what factors affect the probability of T2DM remission after MBS, but also what factors affect T2DM relapse after remission. The aim of this study is to evaluate the success of the modified ABCD score [8] in predicting T2DM relapse following complete remission (CR) after TB and to determine whether MASLD is among the factors affecting T2DM relapse.

## Patients and Methods

Between 2014 and 2019, 454 patients with T2DM were operated by first author. Candidates for primary MBS were defined as having a BMI of at least 32.5 kg/m^2^ and a fasting C-peptide level of at least 1.5 ng/mL. Patients with T2DM who had a BMI of 40 kg/m^2^ or more underwent primary MBS, regardless of whether their fasting C-peptide level was less than 1.5 ng/mL. The choice of procedure was made according to the patient’s preference after the patients were informed about long-term results and complication rates for various procedures. TB surgery was preferred by 97 non-consecutive patients.

**Fig. 1:**
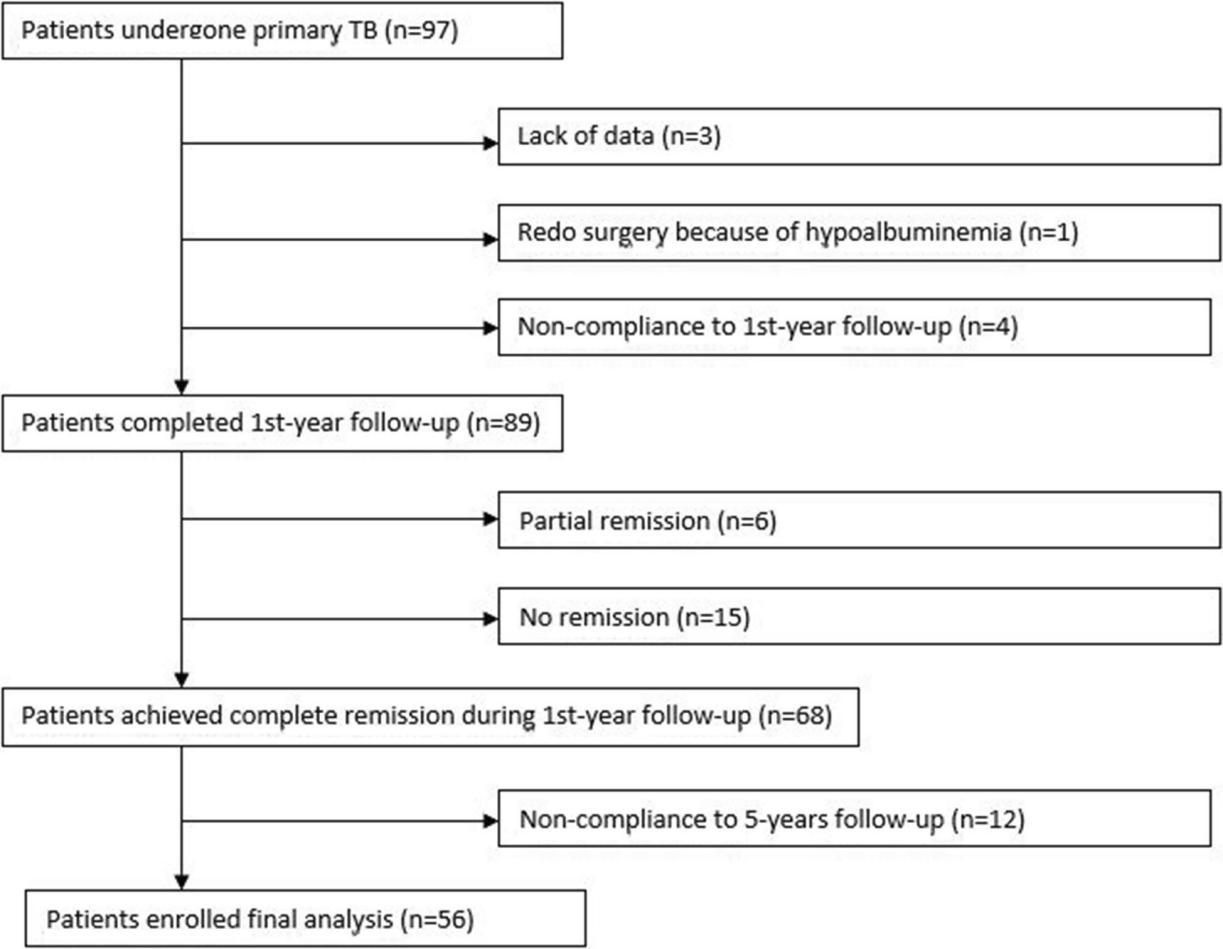
The flow chart of patient enrollment

Bougie size was 40 Fr and stapling started 6 cm proximal to pylorus for sleeve gastrectomy. A 150-cm-long ileal bridge with a 3.5-cm-long gastroileal anastomosis was made using a linear stapler. Until 2018, common channel was 100 cm long, then it was lengthened to 150 cm. The details of follow-up protocol for first year were mentioned in our previous study [5]. The follow-up protocol was repeated twice a year in the second postoperative year and once a year thereafter. The flow chart of patient enrollment is shown in Fig. 1. The data of 56 out of 68 patients who achieved CR following TB were evaluated retrospectively. Ethics committee approval was not obtained due to the retrospective design of the study. The success of the ABCD score in predicting T2DM relapse following CR after TB was the primary outcome of the study. Secondary outcome was to determine whether MASLD was one of the factors contributing to relapse.

Definitions established by the ASMBS Clinical Issues Committee have been used to evaluate the glycemic outcomes of patients after MBS, with the exception of the criterion of recurrence [9]. All cases other than CR, which were defined as “normal measures of glucose metabolism (HbA1c < 6%, FBG < 100 mg/dL) in the absence of antidiabetic medications” were considered relapse, even if there was partial remission (PR). Fibrosis Score 4 (FIB-4 index) was used to assess liver fibrosis due to MASLD. Based on the preoperative and last postoperative serum biomarkers of patients, FIB-4 index was calculated using the formula ([age × aspartate aminotransferase (AST)]/[platelet count × √ alanine aminotransferase (ALT)]). FIB-4 indices were categorized as follows: < 1.3 = low risk, 1.3–2.67 = medium risk, and > 2.67 = high risk for liver fibrosis [10].

### Statistical Analyses

The Kolmogorov–Smirnov test and the Shapiro–Wilk test were used to test the normality of data. Parametric and nonparametric data were presented as mean ± SD and median (min–max), respectively. Chi-square test or Fisher’s exact test was used to assess difference in distribution of categorical variables. While parametric data were compared with *t*-test, nonparametric data were compared with the Mann–Whitney *U* or the Kruskal–Wallis test. For repeated data, the McNemar test or the repeated-measures *t*-test was used. Receiver operating characteristic (ROC) curve analyses were used to determine the area under curve (AUC), and Youden’s index was used to determine the optimal cut-off point for the AUCs. A binary logistic regression analysis was used to analyze the factors affecting relapse. A *p* value less than 0.05 was considered statistically significant. The power of study was 99.9% for exact test and 100% for chi-square test in comparison of patients with and without relapse.

## Results

Of the 56 patients, 29 were female (51.8%). Considering the components of the modified ABCD score, the median age of the patients was 48 years (range: 27 to 73), BMI was 40.8 kg/m^2^ (range: 32.5 to 58.1), C-peptide level was 3.17 ng/mL (range: 1.27 to 6.91), and diabetes duration was 7 years (range: 3 to 17). None of them consumed alcohol and had viral hepatitis. The median ABCD score was 5 (range: 2 to 8). The median follow-up was 71 months (range: 61 to 101) and the follow-up rate of patients who achieved CR in the first postoperative year was 82.4%. The preoperative and postoperative clinical characteristics of the patients are also shown in Table 1.

**Table 1:** Patients characteristics and postoperative outcomes

		Preoperative	Postoperative	*p* value
Sex (F/M)		29/27	/	
Age (years)		48 (27–73)	53 (32–79)	< 0.001
BMI (kg/m^2^)		40.8 (32.5–58.1)	24.8 (19.7–32.7)	< 0.001
HbA1c (%)		9.05 (6.8–13.2)	5.45 (4.6–6.7)	< 0.001
C-peptide (ng/mL)		3.17 (1.27–6.91)	/	NA
Duration of T2DM (years)		7 (3–17)	/	NA
No. of OADs	None	3 (5.4%)	51 (91.1%)	< 0.001
	1	12 (21.4%)	3 (5.4%)	
	2	33 (58.9%)	2 (3.6%)	
	3	8 (14.3%)	0 (0%)	
Insulin treatment	No	14 (25%)	56 (100%)	< 0.001
	Yes	42 (75%)	0 (0%)	
AST (U/L)		30.5 (14–82)	23 (10–32)	< 0.001
ALT (U/L)		33 (13–121)	18 (9–41)	< 0.001
Platelet count (× 10^9^/L)		288.5 (198–453)	283.5 (86–398)	0.012
Albumin (g/dL)		4.26 ± 0.35	4.09 ± 0.42	0.009
Total cholesterol (mg/dL)		200.61 ± 36.80	157.38 ± 44.39	< 0.001
LDL cholesterol (mg/dL)		115.5 (62–211)	97.5 (32–179)	< 0.001
HDL cholesterol (mg/dL)		40 (27–53)	49.5 (32–85)	< 0.001
Triglycerides (mg/dL)		147.5 (71–380)	85 (24–181)	< 0.001
Hyperlipidemia	No	13 (23.2%)	46 (82.1%)	< 0.001
	Yes	43 (76.8%)	10 (17.9%)	
FIB-4 index		0.773 (0.29–2.45)	0.041 (0.01–0.12)	< 0.001
FIB-4 category	Intermediate	8 (14.3%)	0 (0%)	< 0.001
	Low	48 (85.7%)	56 (100%)	

Eleven of 56 patients (19.6%) had relapse in T2DM in follow-up. None of them needed insulin treatment, but five patients resumed oral antidiabetic drugs (OADs). The median time to reinitiating of OADs was 65 months (range: 16 to 84). The remaining six patients were still in PR. BMI, HbA1c level, AST, ALT, total cholesterol, LDL-cholesterol, triglycerides, and FIB-4 index were significantly lower in postoperative period. Additionally, postoperative HDL-cholesterol level was significantly higher than preoperative period. Apart from these favorable outcomes, a reduction in the levels of albumin and platelet count was seen.

**Fig. 2:**
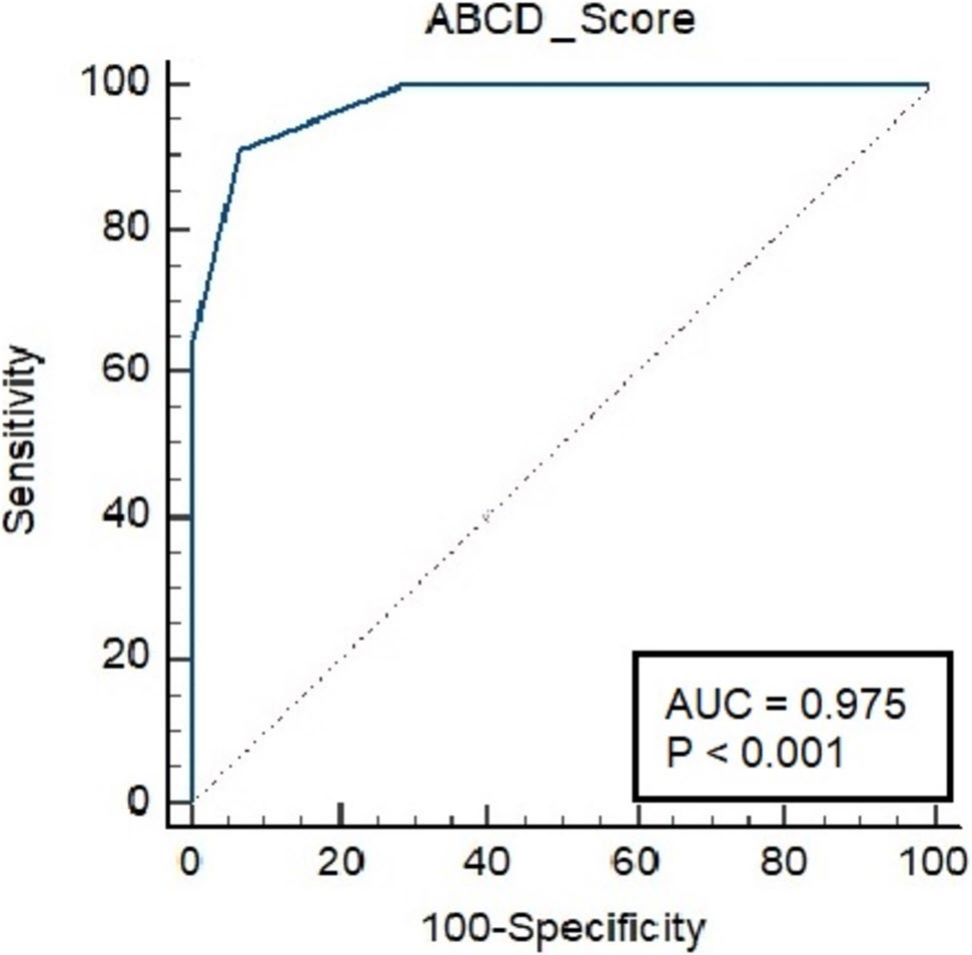
ROC curve analysis of ABCD score for T2DM relapse

In univariate analysis, there were statistically significant differences between patients with and without relapse in the preoperative serum C-peptide and LDL levels, duration of diabetes, ABCD score, and FIB-4 index, and postoperative platelet count (Table 2). According to cut-off point in ABCD score, patients with < 4 points had significantly higher rate of relapse than others (76.9% vs. 2.3%, *p* < 0.001). The ABCD score had sensitivity of 90.9%, specificity of 93.3%, and accuracy of 92.2% based on this cut-off score. The AUC was 0.975 to predict the likelihood of T2DM relapse after TB in patients with an ABCD score less than 4 (Fig. 2). Binary logistic regression analysis showed that the most significant risk predictor for relapse was an ABCD score less than 4 (Table 3). Other preoperative risk factors were lower C-peptide level, higher LDL-cholesterol level, longer DM duration, and higher FIB-4 score.

**Table 2:** Differences in characteristics between patients with and without relapse

			In remission (*n* = 45)	Relapsed (*n* = 11)	*p* value
Preoperative	F/M ratio		22/23	7/4	0.506
	Age		46.13 ± 11.07	51 ± 4.60	0.092
	BMI (kg/m^2^)		41.49 (34.2–52.7)	40 (32.5–58.1)	0.464
	HbA1c (%)		9.5 (6.8–13.2)	8.7 (7.7–9.1)	0.269
	C-peptide (ng/mL)		3.65 (2.17–6.91)	1.6 (1.27–2.79)	< 0.001
	Duration of T2DM (years)		6 (3–15)	12 (7–17)	< 0.001
	ABCD score		5 (3–8)	2 (2–4)	< 0.001
	No. of OADs	None	3	0	0.148
		1	12	0	
		2	24	9	
		3	6	2	
	Insulin treatment	No	13	1	0.258
		Yes	32	10	
	AST (U/L)		31 (14–76)	25 (19–82)	0.642
	ALT (U/L)		39 (13–121)	25 (19–58)	0.577
	Platelet count (× 10^9^/L)		289 (198–453)	288 (206–305)	0.197
	Albumin (g/dL)		4.28 ± 0.35	4.19 ± 0.35	0.437
	Total cholesterol (mg/dL)		199 (130–296)	195 (138–284)	0.265
	LDL cholesterol (mg/dL)		112 (62–190)	148 (73–211)	0.035
	HDL cholesterol (mg/dL)		40.16 ± 6.51	41.09 ± 7.85	0.683
	Triglycerides (mg/dL)		133 (82–380)	182 (71–296)	0.672
	Hyperlipidemia	No	11	2	1.0
		Yes	34	9	
	FIB-4 index		0.71 (0.29–1.64)	0.87 (0.70–2.45)	0.016
	FIB-4 category	Intermediate	5 (%)	3 (%)	0.181
		Low	40 (%)	8 (%)	
Postoperative	BMI (kg/m^2^)		25.8 ± 3.6	25.5 ± 4.8	0.804
	Hba1c (%)		5.33 ± 0.32	6.09 ± 0.41	< 0.001
	AST (U/L)		22 (10–32)	23 (13–26)	0.984
	ALT (U/L)		17 (9–41)	18 (17–26)	0.509
	Platelet count (× 10^9^/L)		291 (151–398)	248 (86–267)	0.008
	Albumin (g/dL)		4.12 ± 0.39	3.95 ± 0.55	0.217
	Total cholesterol (mg/dL)		156.67 ± 41.11	160.27 ± 58.19	0.812
	LDL cholesterol (mg/dL)		87 (51–179)	99 (32–123)	0.490
	HDL cholesterol (mg/dL)		48 (36–85)	56 (32–58)	0.599
	Triglycerides (mg/dL)		85 (24–181)	79 (49–149)	0.613
	Hyperlipidemia	No	37	9	1.0
		Yes	8	2	
	FIB-4 index		0.04 (0.01–0.11)	0.05 (0.02–0.12)	0.348
	FIB-4 category	Intermediate	0 (0%)	0 (0%)	1.0
		Low	45 (100%)	11 (100%)

**Table 3:** Binary logistic regression analysis results for predictors of relapse

Preoperative variables	OR (95% CI)	*p* value
Sex (F/M)	1.830 (0.469–7.131)	0.384
Age (years)	1.048 (0.981–1.119)	0.164
BMI (kg/m^2^)	0.985 (0.886–1.094)	0.772
HbA1c (%)	0.711 (0.435–1.160)	0.172
C-peptide (ng/mL)	0.032 (0.003–0.295)	0.002
AST (U/L)	1.019 (0.986–1.053)	0.265
ALT (U/L)	0.983 (0.955–1.012)	0.248
Platelet count (× 10^9^/L)	0.990 (0.978–1.003)	0.128
Albumin (g/dL)	0.460 (0.067–3.173)	0.431
Total cholesterol (mg/dL)	0.995 (0.977–1.014)	0.634
LDL cholesterol (mg/dL)	1.025 (1.005–1.045)	0.013
HDL cholesterol (mg/dL)	1.021 (0.925–1.127)	0.677
Triglycerides (mg/dL)	1.003 (0.995–1.011)	0.454
Hyperlipidemia (no/yes)	0.687 (0.128–3.672)	0.661
DM duration (years)	1.553 (1.216–1.983)	< 0.001
No. of OADs	2.688 (0.896–8.067)	0.078
Insulin treatment (no/yes)	0.246 (0.029–2.122)	0.202
ABCD score	0.047 (0.006–0.361)	0.003
Having an ABCD score less than 4	140.0 (13.141–1491.529)	< 0.001
FIB-4 index	6.073 (1.496–24.656)	0.012
FIB-4 category	3.000 (0.594–15.162)	0.184

## Discussion

The results of present study suggest that higher risk for T2DM relapse following TB is associated with longer duration of diabetes, low C-peptide level indicating a decrease in pancreatic beta cell functions, and higher LDL-cholesterol level and FIB-4 index which may indicate deterioration in the metabolic functions of the liver. Relapse is more common in patients whose ABCD score is less than 4.

Although it is now widely known that T2DM might recur after an initial remission following MBS [6, 11], the precise incidence of this phenomenon is hard to understand because most studies report early results and do not focus on relapse. Furthermore, many studies do not address the severity of diabetes, and different MBS techniques have varying effectiveness in correcting the underlying metabolic disorder. Early relapse has been associated with a number of clinical variables, including poor preoperative diabetes control, longer duration of the disease, insulin requirement for glucose control, and decreased pancreatic beta cell reserve [6, 11, 12]. The number of antidiabetic medications is another indicator that might suggest the severity of diabetes. Aminian et al. reported that it was an independent risk factor for late relapse [7]. They also suggested that less weight loss in the first postoperative year and greater weight regain were also independent risk predictors. Additionally, Jans et al. proposed that women are at higher risk [13]. The present study demonstrated that longer duration of diabetes and poor beta cell reserve are risk factors as consistent with earlier researches. Neither gender nor the quantity of antidiabetic medications used is an independent predictor of late T2DM relapse in this cohort. This might be because study participants were not receiving adequate antidiabetic therapy. The high preoperative median Hba1c level supports this possibility.

Beyond these risk factors, fibrosis in the liver due to MASLD was associated with higher risk for relapse in the present study. The increasing amount of evidence indicates that MASLD may either help or hinder the development of T2DM, and that there is a relationship between the severity of MASLD and the risk of developing T2DM [14]. To date, however, there has not been any research done on the connection between severity of MASLD and T2DM relapse following MBS. Although liver biopsy is the reference standard for the diagnosis of MASLD, it has limitations as it is an invasive procedure and is not suitable for population screening [10]. Therefore, some diagnostic tools have been developed that predict the stage of fibrosis in liver, such as NFS and FIB-4 index, based on various serum biomarkers. These have different cut-off values in various clinical presentations, such as alcoholic liver disease, non-alcoholic liver disease, viral infections affecting the liver, and have been validated in MASLD that our study cohort possesses [15]. There is no better method to define liver fibrosis in the present cohort because there is no sufficient number of liver biopsies taken from patients with T2DM undergoing TB or preoperative elastosonography data.

It is well documented that there are differences in the potency of various kinds of MBS for treating T2DM [1]. As a result, it is reasonable to assume that there will be variations in the frequency of relapse following various surgical procedures. Arterburn et al. analyzed 2254 people with T2DM who had initial remission after RYGB and found that 35% of them had a relapse [6]. Aminian et al. reported that patients who had RYGB surgery had a 30% relapse rate, whereas those who had sleeve gastrectomy had a 43% rate in a median 8-year follow-up [7]. In contrast, Jans et al. found no significant relationship between relapse and the kind of surgery the patients underwent (SG or RYGB) and a lower relapse rate of 9.5% during a 5-year follow-up [13]. With the exception of Aminian et al., no one has categorized diabetes based on severity and suggested the type of MBS based on the likelihood that the patient may experience a relapse in long-term follow-up [16]. Evidence-based selection of metabolic surgery for T2DM based on disease severity is logical. Therefore, data and research that assess relapse rates in long-term follow-up for various surgical procedures based on the severity of the disease must be gathered and published in the literature. To date, no study has been published investigating T2DM relapse after TB.

The present research revealed that relapse rate of T2DM after TB was 19.6% and that up to 90% of relapses occurred in patients with an ABCD score below 4, which can be described as severe diabetes. This rate could appear high at first. In fact, if we had used the diabetes diagnostic criteria defined by the American Diabetes Association (ADA) Professional Practice Committee in 2024 [17], we would not have accepted six patients in partial remission as relapses. However, since this study is a continuation of our previous study, we preferred to use ASMBS criteria instead of ADA criteria. Even though some patients experienced relapse in the long term, the significant improvement in the metabolic profile after TB continued.

It is important to recognize the limitations of this study. The main limitations of this study are the retrospective design, the small number of patients, the limited follow-up, and the bias caused by patients lost during follow-up. Although a retrospective design has obvious disadvantages, no prospective large study exclusively concentrating on the relapse of T2DM after TB has been published in the scientific literature due to the challenges of prospectively following up any large cohort over an extended period of time. When the rate of TB among MBS methods performed all over the world is considered, it becomes more evident how difficult it is to collect prospective data involving a large number of patients. Another issue with this study is its limited follow-up, but a median follow-up of 71 months (with at least 5 years’ follow-up for every patient) in such a cohort with limited knowledge in the literature would still be considered acceptable.

Despite its limitations, this study remains valuable because it is the first study to report the long-term risk of relapse in patients with TB by correlating it with the severity of T2DM in the preoperative period. Since almost all relapses occur in patients with an ABCD score below 4, this group of patients should be monitored more closely. Since one of the important findings of this study is that there is a relationship between liver fibrosis and T2DM relapse, elastosonography or intraoperative liver biopsy may be recommended in patients with T2DM to evaluate liver fibrosis. Thus, liver fibrosis can be more precisely assessed, and patients with higher fibrosis can be warned to choose proven more efficient anti diabetic surgical techniques and to remain under closer follow-up.

## Conclusion

The ABCD score has 90.9% sensitivity and 93.3% specificity in predicting T2DM relapse after achieving initial CR of T2DM with TB if it is below 4 points. Patients with longer durations of T2DM, higher LDL-cholesterol levels, lower C-peptide levels and ABCD scores, and worse liver fibrosis are at a higher risk of relapse. Patients with these characteristics should be warned that they have a high risk of relapse.

## Data Availability

No datasets were generated or analysed during the current study.

## References

[1] Kodama S, Fujihara K, Horikawa C, et al. Network meta-analysis of the relative efficacy of bariatric surgeries for diabetes remission. Obes Rev. 2018;19:1621–9. 10.1111/obr.12751. 30270528 10.1111/obr.12751

[2] Plaeke P, Beunis A, Ruppert M, et al. Review, performance comparison, and validation of models predicting type 2 diabetes remission after bariatric surgery in a Western European population. Obes Surg. 2021;31:1549–60. 10.1007/s11695-020-05157-0. 33398626 10.1007/s11695-020-05157-0

[3] Ballestri S, Zona S, Targher G, et al. Nonalcoholic fatty liver disease is associated with an almost twofold increased risk of incident type 2 diabetes and metabolic syndrome. Evidence from a systematic review and meta-analysis. J Gastroenterol Hepatol. 2016;31:936–44. 10.1111/jgh.13264. 26667191 10.1111/jgh.13264

[4] Santoro S, Castro LC, Velhote MC, et al. Sleeve gastrectomy with transit bipartition: a potent intervention for metabolic syndrome and obesity. Ann Surg. 2012;256:104–10. 10.1097/SLA.0b013e31825370c0. 22609843 10.1097/SLA.0b013e31825370c0

[5] Kirkil C, Aydin I, Yur M, Ag O, Bozcan MY. Comparison of the ABCD Score's accuracy in predicting remission of type 2 diabetes mellitus one year after sleeve gastrectomy, one anastomosis gastric bypass, and sleeve gastrectomy with transit bipartition. Obes Surg. 2024;34:133–140. 10.1007/s11695-023-06950-3. 10.1007/s11695-023-06950-3 37985569

[6] Arterburn DE, Bogart A, Sherwood NE, et al. A multisite study of long-term remission and relapse of type 2 diabetes mellitus following gastric bypass. Obes Surg. 2013;23:93–102. 10.1007/s11695-012-0802-1. 23161525 10.1007/s11695-012-0802-1 PMC4641311

[7] Aminian A, Vidal J, Salminen P, et al. Late relapse of diabetes after bariatric surgery: not rare, but not a failure. Diabetes Care. 2020;43:534–40. 10.2337/dc19-1057. 31974105 10.2337/dc19-1057

[8] Lee WJ, Almulaif A, Tsou JJ, et al. Laparoscopic sleeve gastrectomy for type 2 diabetes mellitus: predicting the success by ABCD score. Surg Obes Relat Dis. 2015;11:991–6. 10.1016/j.soard.2014.12.027. 25868836 10.1016/j.soard.2014.12.027

[9] Brethauer SA, Kim J, El Chaar M, et al, ASMBS Clinical Issues Committee Standardized outcomes reporting in metabolic and bariatric surgery. Obes Surg. 2015;25:587–606. 10.1007/s11695-015-1645-3 10.1007/s11695-015-1645-3 25802064

[10] Rinella ME, Neuschwander-Tetri BA, Siddiqui MS, et al. AASLD Practice Guidance on the clinical assessment and management of nonalcoholic fatty liver disease. Hepatology. 2023;77:1797–835. 10.1097/HEP.0000000000000323. 36727674 10.1097/HEP.0000000000000323 PMC10735173

[11] Andalib A, Aminian A. Sleeve gastrectomy and diabetes: is cure possible? Adv Surg. 2017;51:29–40. 10.1016/j.yasu.2017.03.003. 28797344 10.1016/j.yasu.2017.03.003

[12] Debédat J, Sokolovska N, Coupaye M, et al. Long-term relapse of type 2 diabetes after Roux-en-Y gastric bypass: prediction and clinical relevance. Diabetes Care. 2018;41:2086–95. 10.1016/10.2337/dc18-0567. 30082327 10.2337/dc18-0567

[13] Jans A, Szabo E, Näslund I, et al. Factors affecting relapse of type 2 diabetes after bariatric surgery in Sweden 2007–2015: a registry-based cohort study. Surg Obes Relat Dis. 2022;18:305–12. 10.1016/j.soard.2021.12.005. 34974997 10.1016/j.soard.2021.12.005

[14] Targher G, Corey KE, Byrne CD, et al. The complex link between NAFLD and type 2 diabetes mellitus — mechanisms and treatments. Nat Rev Gastroenterol Hepatol. 2021;18:599–612. 10.1038/s41575-021-00448-y. 33972770 10.1038/s41575-021-00448-y

[15] Sugiyama A, Kurisu A, Bunthen E, et al. Distribution of FIB-4 index in the general population: analysis of 75,666 residents who underwent health checkups. BMC Gastroenterol. 2022;22:241. 10.1186/s12876-022-02290-1. 35562658 10.1186/s12876-022-02290-1 PMC9101936

[16] Aminian A, Brethauer SA, Andalib A, et al. Individualized metabolic surgery score: procedure selection based on diabetes severity. Ann Surg. 2017;266:650–7. 10.1097/SLA.0000000000002407. 28742680 10.1097/SLA.0000000000002407

[17] American Diabetes Association Professional Practice Committee. 2. Diagnosis and classification of diabetes: standards of care in diabetes-2024. Diabetes Care. 2024;47(Suppl 1):S20-S42. 10.2337/dc24-S002. 10.2337/dc24-S002 PMC10725812 38078589

